# Combination of electromagnetic navigation bronchoscopy‐guided microwave ablation and thoracoscopic resection: An alternative for treatment of multiple pulmonary nodules

**DOI:** 10.1111/1759-7714.13443

**Published:** 2020-04-14

**Authors:** Ning Jiang, Liming Zhang, Yingtao Hao, Xiaolin Wu, Yunpeng Zhao, Bo Cong, Chuanliang Peng

**Affiliations:** ^1^ Department of Thoracic Surgery The Second Hospital of Shandong University Jinan China; ^2^ Key Laboratory of Chest Cancer Shandong University Jinan China; ^3^ Department of Pathology The Second Hospital of Shandong University Jinan China

**Keywords:** Early stage lung cancer, electromagnetic navigation bronchoscopy, microwave ablation, pulmonary nodules

## Abstract

Here, we describe a novel method using microwave ablation (MWA) guided by electromagnetic navigation bronchoscopy (ENB) and video‐assisted thoracoscopic surgery (VATS) for simultaneous treatment of multiple high‐risk pulmonary nodules in a 47‐year‐old woman. After the ENB registration process, the operator delivered the locatable electromagnetic probe to the target in the right upper lobe following the navigational route. MWA was performed after an antenna was passed into the lesion through the working channel. The wedge resection of the left upper lobe and lower lobe and the lingual segment resection were performed by VATS. The pathological diagnoses was adenocarcinoma in situ (AIS) of the right upper lobe lesion, AIS of the left upper lobe, minimally invasive adenocarcinoma of the left lower lobe lesion and chronic inflammation of the lingular segment. MWA guided by ENB combined with VATS is an alternative treatment strategy to deal with multiple pulmonary nodules at the same stage of the operation.

## Introduction

With the increasing influence of early lung cancer screening programs and the use of low‐dose computed tomography (LDCT), the detection rate of pulmonary nodules has climbed from 0.2% to 40%–60%.^1^ These nodules are ground‐glass opacity (GGO) or ground‐glass nodule (GGN), and approximately 20% of GGNs may be increased in size or found to have solid components in the follow‐up period.^2^ A number of these GGNs are early stage lung cancer with pathology consistent with adenocarcinoma in situ (AIS) or minimally invasive adenocarcinoma (MIA). High‐risk GGNs, with increases in the size or solid components, require surgical intervention.^2^

It is easier to deal with a single pulmonary nodule diagnosed as early stage lung cancer by minimally invasive thoracoscopic surgery, including lobectomy, wedge resection and segmentectomy. The difficulty of the current treatment strategy is that multiple pulmonary nodules cannot be definitively diagnosed as benign or malignant. In particular, the need for surgical intervention is a challenge for patients with multiple nodules suspected or confirmed to be malignant. These multiple high‐risk pulmonary nodules in the same side could be surgically removed at the same stage, and those on both sides could not easily be removed, as is typical.

To find a more minimally invasive and effective treatment, thermal ablation therapy has been used in patients with pulmonary nodules, including radiofrequency ablation (RFA) and microwave ablation (MWA). Bleeding, pneumothorax, intrathoracic hemorrhage and bronchothoracic fistulas may be the most serious complications of percutaneous thermal ablation treatment.

Electromagnetic navigation bronchoscopy (ENB) can be directed to any position of the full lung through the natural cavity, and an electromagnetic probe be directed at a more accurate position in the pulmonary lesion through electromagnetic navigation. This feature of ENB provides the foundation for the possible treatment of pulmonary diseases.

For patients with multiple pulmonary nodules, a more minimally invasive treatment than thoracoscopic surgery may involve ablation therapy through ENB. In the case reported here, we used a novel technology in which MWA was guided by ENB in combination with VATS to treat a patient with multiple pulmonary nodules on both sides of the lung.

## Case report

**Figure 1:**
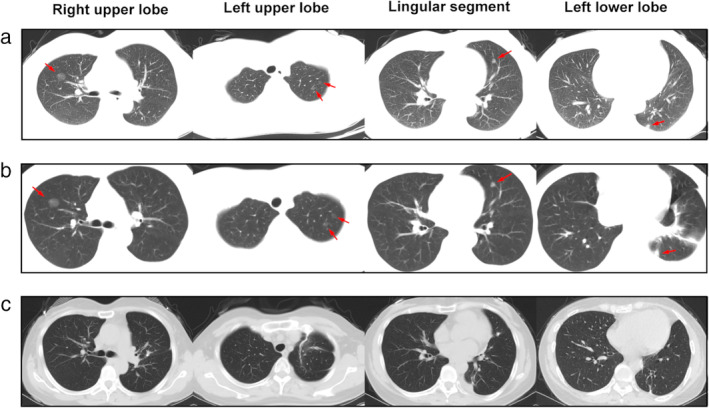
(**a**) CT image after trauma showed a pure ground‐glass nodule (pGGN) of approximately 14 mm in the right upper lobe, two pGGNs of approximately 6 mm in the apical segment of the left upper lobe, a mixed GGN (mGGN) of approximately 8 mm in the left lingular segment, and a mGGN of approximately 8 mm in the left lower lobe. (**b**) There were no significant changes in the nodules after one week of anti‐inflammatory treatment. (**c**) The pGGN in right upper lobe had disappeared. Shadows of staples and postoperative changes can be seen in the left upper and lower lobes.

A 47‐year‐old female with chest trauma and fractured ribs was diagnosed with multiple pulmonary nodules following a chest computed tomography (CT) examination. CT imaging (Fig 1a) revealed a 14 mm pure ground‐glass nodule (pGGN) in the right upper lobe, two 6 mm pGGNs in the apical segment, an 8 mm mixed GGN (mGGN) in the lingular segment, and an 8 mm mGGN in the left basal segment without enlargement of the hilar or mediastinal lymph nodes. Lung cancer‐related biomarkers carcinoembryonic antigen (CEA), neuron specific enolase (NSE) and cytokeratin fragment antigen 21‐1 (CYFRA21‐1) levels were within normal limits.

We highly suspected that these lesions were early stage lung cancer according to the radiologic characteristics. Antibiotics were used for the treatment of sputum because of the chest trauma and rib fracture. The nodules did not shrink or disappear after one week of anti‐inflammatory treatment (Fig 1b). In the International Early Lung Cancer Action Program protocol (I‐ELCAP),^3^ a follow‐up CT within 1–3 months is recommended after antibiotic treatment to determine if the largest noncalcified nodules (≥15 mm) are probably an infection according to baseline screening. The purpose of a CT scan was to assess whether there were any complications due to trauma, such as infection, hemothorax and pneumothorax. We did not expect the nodules to disappear completely after one week of antibiotic treatment, as this was not enough time to treat pulmonary infection.

Fortunately, no serious complications of chest trauma were found in the follow‐up CT scan. However, there were also no changes in the pulmonary nodules according to the CT imaging. Considering the stable condition of the trauma and that the lesions may have been indolent adenocarcinoma or chronic lesions, we recommended a CT scan after three months, and the patient was discharged from the hospital. The patient did not accept a follow‐up CT at three months because the multiple pulmonary nodules made her very anxious, afraid and affected her sleep. She requested simultaneous treatment of the nodules on both sides and accepted the surgical plan. After obtaining informed consent, we performed MWA in the right lung guided by ENB and performed VATS in the left lung at the same stage.

VATS may difficult to perform if there is no localization of the GGNs. In this case, we determined the target of the proposed resection by three‐dimensional reconstruction with CT imaging before the surgical procedure. We decided to perform segmentectomy because of the GGNs located within the deep location in the lingular segment. Wedge resections were planned to be performed since the GGNs in the upper and lower lobe were near the edge of the lung. We imported the CT images into SuperDimension software (SuperDimension Inc., Minneapolis, MN, USA) and created an operation plan before commencing the ENB procedure.

**Figure 2:**
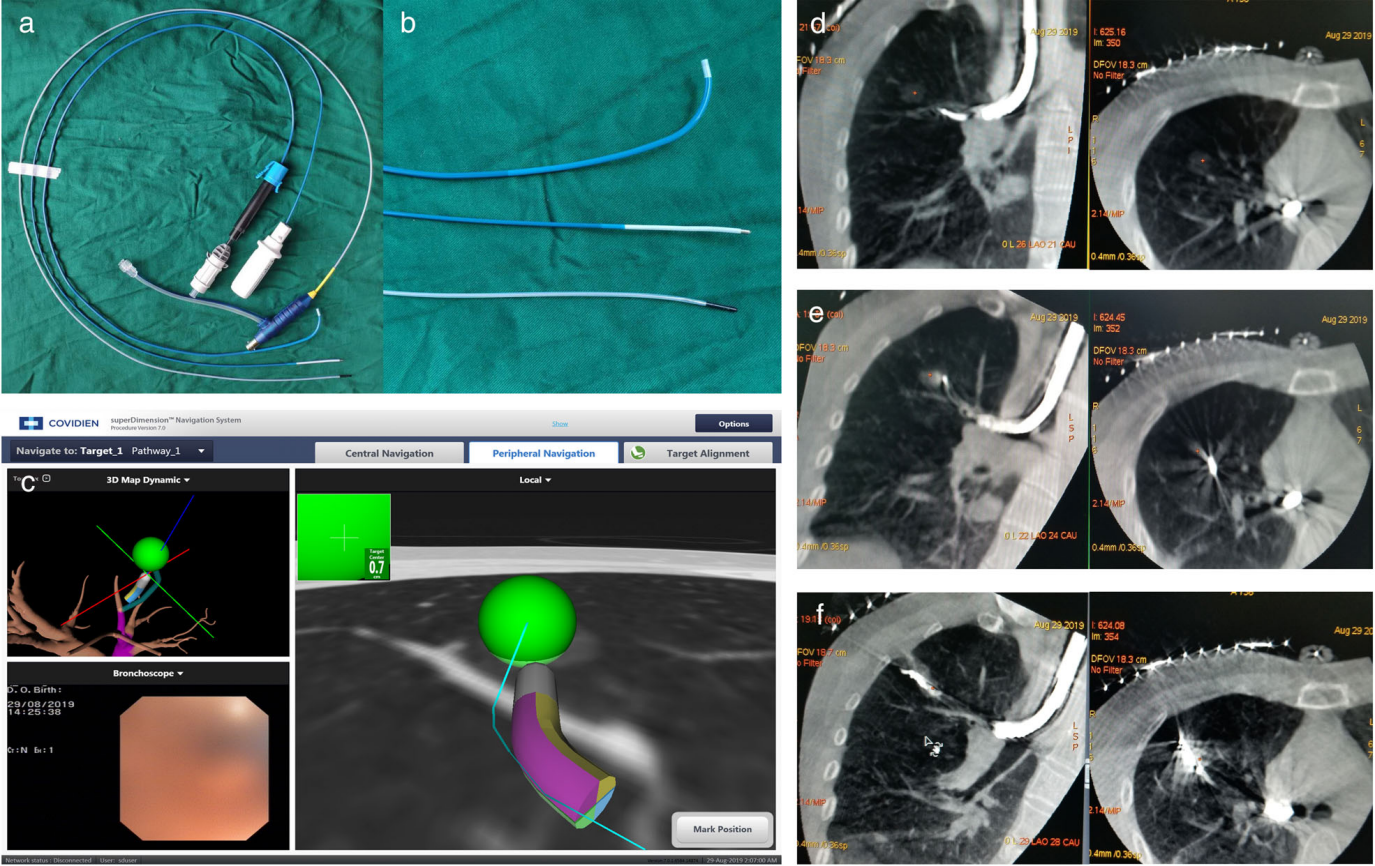
(**a**) The devices used for microwave ablation (MWA) guided by electromagnetic navigation bronchoscopy (ENB) including the ENB working channel catheter (curved‐tip catheter), magnetic probe and customized microwave antenna (with black short needle head), respectively. (**b**) Magnified image of the three devices. (**c**) ENB image shows that the working channel catheter has accurately reached a position adjacent to the lesion by guidance of the magnetic probe. MWA guided by ENB in CBCT image. (**d**) The magnetic probe and working channel catheter were inserted into the bronchus through the bronchoscope. (**e**) CT image shows that the antenna arrived at the lesion through the working channel catheter before MWA commenced. (**f**) After MWA was guided by ENB, the area and density of the shadow increased in the location of the lesion.

The patient was placed in a supine position under general anesthesia. Once registration was satisfactory, the bronchoscope was placed into the right upper lobar bronchus. The working channel catheter guided by the sensing device arrived at the lesion through the bronchoscope. The ENB image showed the location of the magnetic probe and working channel catheter adjacent to the lesion (Fig 2c).

**Figure 3:**
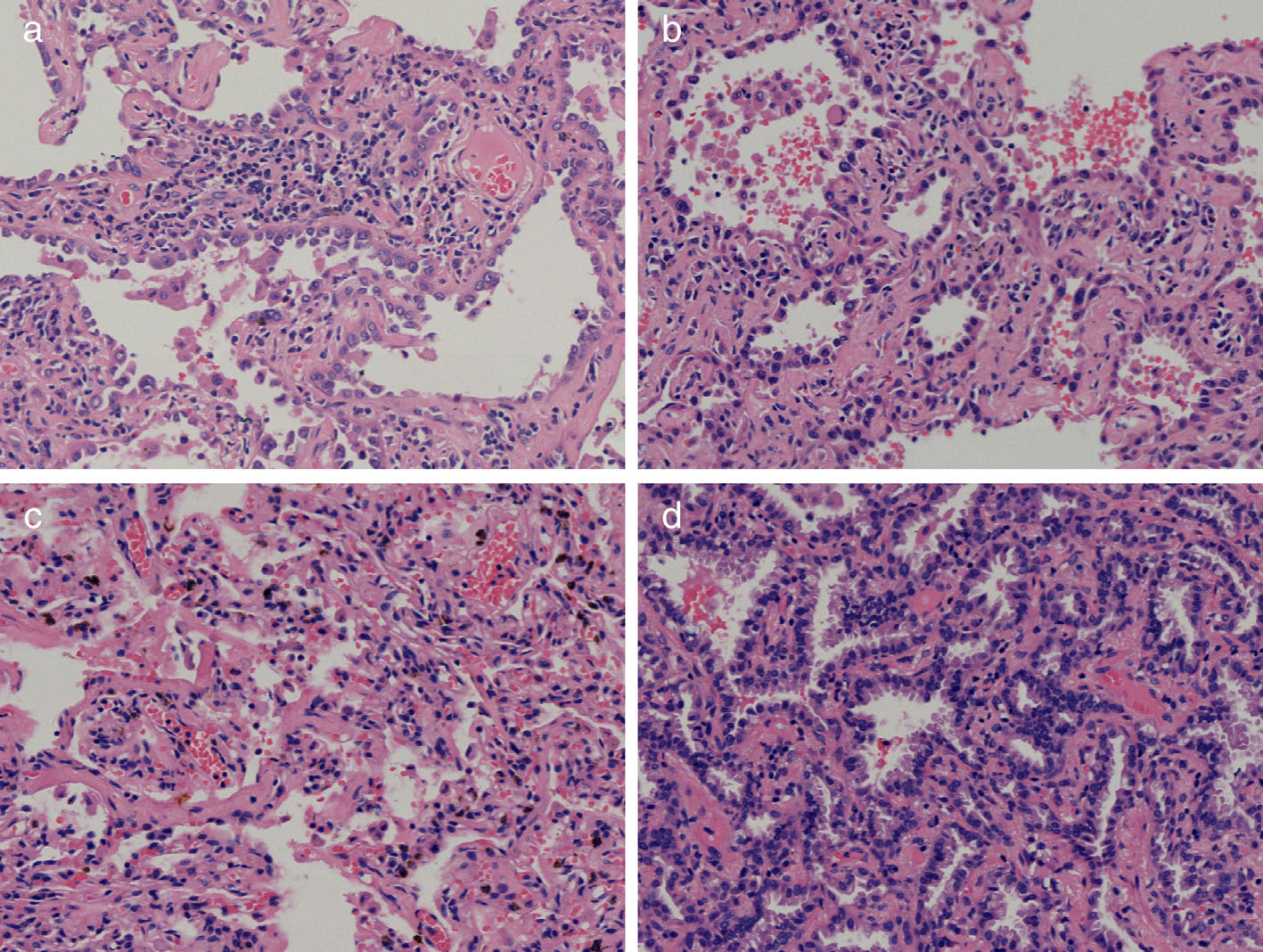
Pathology of the pulmonary nodules (HE stain, 20×). (**a**) The nodule in the right upper lobe was adenocarcinoma in situ (AIS). (**b**) The two nodules in the left upper lobe were AIS. (**c**) The nodule in the lingular segment was chronic inflammation. (**d**) The nodule in the left lower lobe was confirmed to be minimally invasive adenocarcinoma (MIA).

We obtained the lesion tissue with a biopsy tool for pathological diagnosis through the working channel. The model of the microwave generator was the same as that used for routine clinical applications. A new shape for the microwave antenna was designed, since we wanted the microwave antenna to arrive at the target through the working channel (2.0 mm in diameter) of the ENB. By removing the long needle and handle, the microwave antenna (1.8 mm in diameter, Fig 2a and b) was customized with a short needle head and a long wire tail (1.8 mm in diameter), which was connected to the generator. After the frozen pathology report determined a diagnosis of AIS (Fig 3a), the MWA antenna was inserted through the working channel and was confirmed to arrive at the presupposed location with the assistance of Cone Beam CT. The CBCT image showed the location of the antenna, which met the treatment needs (Fig 2e). MWA treatment was performed with an output power of 52 W for 10 minute by using a 2.45 GHz microwave generator (AMICA‐GEN, HS Hospital Service, Aprilia, Italy). A CBCT scan was again obtained to confirm the range of the ablation. The CT imaging showed that the shadow of the treatment region was larger in diameter and higher in density (Fig 2f) than the pretreatment GGN lesion. This indicated that the ablation covered the lesion completely.

After the MWA treatment was completed, a video‐assisted thoracoscopic surgery (VATS) was carried out for resection of the left lung lesions. Placing the patient in a right lateral decubitus position, we performed a 1 cm skin incision on the posterior axillary line in the seventh intercostal space, through which a 10 mm trocar for a thoracoscope (Karl Storz GmbH & Co) was inserted. A 3 cm incision for surgical devices was subsequently created, and a wound retractor inserted at the anterior axillary line in the fourth intercostal space. No transfer of pleural nodules or pleural effusion was found in the chest. The vein, artery and bronchia of the lingular segment were dissected and cut off, respectively. Expanding and collapsing the lung to confirm the intersegmental plane of the lingular segment, we cut out the lingular segment using Echelon Flex Endopath staplers (Ethicon, USA). Wedge resection was performed for the upper and lower lobe nodules. The mediastinal and hilar lymph nodes were resected on the basis of the frozen pathology results during surgery.

The pathological diagnoses of the nodules in the left upper lobe, lingular segment and lower lobe were AIS, chronic inflammation and MIA, respectively (Fig 3b–d). The N1 and N2 station lymph nodes that were sampled were all negative. The histologic results indicated that the range of resection was satisfactory for the treatment required.

The total volume of the left thoracic drainage was approximately 120 mL postoperatively. The thoracic drainage tube was removed on the second day after surgery. There were no complications such as fever, hemoptysis, pneumothorax or infection. The patient was discharged from hospital upon recovery on the fourth day after surgery.

At three month follow‐up, a CT scan was obtained to evaluate the treatment effect. The increased shadow after MWA had disappeared from the CT image (Fig 1c), which indicated that the necrotic area generated by MWA had been completely absorbed. The high‐density shadows in the left upper and lower lobe were traces of staples. A longer follow‐up was recommended for this patient to observe the long‐term treatment effect.

## Discussion

Lung cancer is among the most common malignant tumors worldwide and has the highest morbidity and mortality. Lung cancer screening through low‐dose chest CT decreases the mortality of the tumor by approximately 20% because of early diagnosis.^4^ With the increasing usage of LDCT in health examination, an increasing number of small pulmonary nodules are being identified in healthy people.

Many research studies have reported management and treatment strategies for pulmonary nodules, including pGGO, mGGO and solid GGN.^5^, ^6^ NCCN guidelines recommend^5^ that for incidental discovery of solid nodules (≥6 to <8 mm), CT should be repeated between 6–12 months. If there are solid nodules ≥8 mm, a repeat CT at three months, PET‐CT or biopsy are recommended. Incidental subsolid nodules contain pGGN and partially solid nodules. The guidelines recommend CT at 6–12 months (≥6 mm) to confirm that there is no growth or change in the solid components of pGGN and then recommend a CT scan is performed every two years until five years. For partially solid nodules, CT is recommended at 3–6 months to confirm there is no change in the solid component, and then annual CT is recommended for five years. If the solid component ≥6 mm, PET‐CT or biopsy is recommended. For multiple subsolid nodules, it is recommended that the CT scan should be repeated at 3–6 months and then subsequent management based on the most suspicious nodule should be provided. Surgical resection is still the first choice for early stage lung cancer. Thermal ablation is an option for selected patients who are medically inoperable.

The British Thoracic Society guidelines^7^ recommend a nodule size threshold for follow‐up (≥5 mm or ≥ 80 mm^3^) and reduction of the follow‐up period to one year for solid nodules. Reassessment of subsolid nodules (≥5 mm) by CT has been recommended at three months, and the risk of malignancy should be calculated. They provide two malignancy prediction calculators to evaluate the risk of lung cancer in nodules. The volume doubling time (VDT) should be calculated based on follow‐up CT at three months and one year to assess the growth of nodules ≥80 mm^3^, or with a ≥6 mm maximum diameter and based on follow‐up CT at one year for nodules ≥5 to <6 mm. According to the VDT and the risk of malignancy, observation and management including repeat CT, PET‐CT, biopsy, resection or nonsurgical treatment were offered to the appropriate persons. For persons who are unfit for surgery and have a high probability of malignancy, stereotactic ablative body radiotherapy (SABR) or radiofrequency ablation (RFA) could be considered if the biopsy is nondiagnostic or impossible. Sublobar resection such as wedge resection and segmentectomy has been suggested to be a feasible surgical method for cT1N0 lung adenocarcinoma.^8^

Multiple pulmonary nodules present challenges for management and clinical treatment. The original Fleischner Society guidelines and the NCCN lung cancer guidelines^5^, ^9^, ^10^ recommend management of multiple pulmonary nodules based on risk factors. By referencing the size, morphological characteristics and risk level, the most suspicious nodule is proposed to be used as the guide for management, including follow‐up, biopsy or surgery.

Nodules considered as malignant by follow‐up or biopsy need further treatment. Multiple suspect primary early stage lung cancer pulmonary nodules can be removed through thoracoscopic minimally invasive surgery. The removal of ipsilateral pulmonary nodules requires the patients to be in relatively good physical health. However, a higher level of physical health is required for patients to undergo the bilateral lung operation. Some early stage lung cancer patients do not qualify for surgical therapy because of poor general condition, although surgery is the best treatment for this type of cancer.

Thermal ablation is a technology that destroys local tissue by transferring energy and producing heat in excess of 50°C. Heat can be produced by various forms of energy, including microwave, radiofrequency, and lasers.^11^ Microwaves are electromagnetic waves with frequencies between 300 MHz and 300 GHz. The oscillation of positively and negatively charged water molecules caused by microwaves results in the heating of local tissue.^12^ Radiofrequencies create heat by resistive heating when an electrical current passes through the ionic components of tissue. Compared to RFA, MWA creates heat more rapidly because it is not affected by tissue carbonization and dehydration. In addition, MWA is less susceptible to the heat‐sink effect of peritumoral vessels.^13^

CT image‐guided thermal ablation therapies, including radiofrequency (RFA), cryoablation and microwave (MWA) therapies, offer potentially curative therapeutic opportunities for those early stage lung cancer patients who are unable to tolerate surgery. MWA used for clinical treatment was first reported in the late 1970s^14^ and was widely used in the treatment of early stage lung cancer patients in 2008,^15^ which was confirmed to have comparable clinical outcomes to those of RFA.^16^ MWA can be used only in stage I NSCLC with a tumor diameter less than 3 cm and no lymph node metastasis.^17^, ^18^ Percutaneous ablation may cause serious complications such as intrathoracic hemorrhage, pneumothorax and bronchopleural fistula.

Electromagnetic navigation bronchoscopy was first used for biopsy of pulmonary nodules that were difficult to reach with conventional bronchoscopy. In recent years, ENB has also been used for preoperative localization of small malignant pulmonary nodules which are difficult to detect during VATS.^19^ ENB should have more application in clinical treatment, which should not be limited to biopsy and localization.

New therapeutic technologies offer lung cancer patients more options. In our study, we investigated a new use for ENB in the treatment of pulmonary nodules. Considering that bilateral nodules are both high risk malignant lesions, the remaining lesions will cause difficulty during follow‐up treatment no matter which side is surgically treated first. Since there is a high risk associated with bilateral surgery, MWA guided by ENB combined with VATS was performed for treatment of the patient with bilateral pulmonary nodules. We obtained pathological diagnosis of the right upper lobe GGO by ENB to determine the indication for MWA and assess the feasibility of contralateral surgery. The energy of MWA caused the lesion and surrounding tissue to undergo degeneration and necrosis. The changes in the CT image after treatment showed a more extensive and deeper shadow than previously observed. Three months later, at a subsequent visit, the chest CT scan revealed the postoperative shadow had been completely absorbed.

Since the nodule in the right lung was diagnosed as AIS and the treatment was satisfactory, the nodules in the left lung were removed by VATS during the concurrent treatment. The postoperative pathology of these nodules was confirmed to be two AIS of the left upper lobe, chronic inflammation of the lingular segment and MIA of the left lower lobe with no lymph node metastasis. This therapy with combined techniques demonstrates superior minimally invasive features with the advantages of reduced postoperative pain and rapid recovery. Theoretically, this patient will have an improved prognosis and require long‐term follow‐up.

MWA guided by ENB instead of percutaneous treatment showed a good short‐term result outcome in our case. This technique may be more suitable for the treatment of multiple pulmonary nodules, even though we only treated one lesion in the right lung. At present, we strictly select the adaptive signs of treatment, especially the pathological types. A larger scale and longer follow‐up study should be developed and implemented to confirm the effect of this treatment regimen. In the future, MWA guided by ENB may be a potential solution instead of surgery for patients with multiple pulmonary nodules.

## Disclosure

None of the authors have any potential conflicts of interest.
